# Potential Impact of Sexual Transmission on Ebola Virus Epidemiology: Sierra Leone as a Case Study

**DOI:** 10.1371/journal.pntd.0004676

**Published:** 2016-05-02

**Authors:** Jessica L. Abbate, Carmen Lia Murall, Heinz Richner, Christian L. Althaus

**Affiliations:** 1 Institute for Ecology and Evolution, University of Bern, Bern, Switzerland; 2 UMR MIVEGEC (UMR CNRS 5290, IRD 224, UM), Institute for Research of Development (IRD), Montpellier, France; 3 UMR UMMISCO (UMI 209 IRD-UPMC), Bondy, France; 4 Max-Planck Institute for Dynamics and Self-Organization, Gottingen, Germany; 5 Institute of Social and Preventive Medicine (ISPM), University of Bern, Bern, Switzerland; Centers for Disease Control and Prevention, UNITED STATES

## Abstract

**Background:**

Sexual transmission of Ebola virus disease (EVD) 6 months after onset of symptoms has been recently documented, and Ebola virus RNA has been detected in semen of survivors up to 9 months after onset of symptoms. As countries affected by the 2013–2015 epidemic in West Africa, by far the largest to date, are declared free of Ebola virus disease (EVD), it remains unclear what threat is posed by rare sexual transmission events that could arise from survivors.

**Methodology/Principal Findings:**

We devised a compartmental mathematical model that includes sexual transmission from convalescent survivors: a SEICR (susceptible-exposed-infectious-convalescent-recovered) transmission model. We fitted the model to weekly incidence of EVD cases from the 2014–2015 epidemic in Sierra Leone. Sensitivity analyses and Monte Carlo simulations showed that a 0.1% per sex act transmission probability and a 3-month convalescent period (the two key unknown parameters of sexual transmission) create very few additional cases, but would extend the epidemic by 83 days [95% CI: 68–98 days] (p < 0.0001) on average. Strikingly, a 6-month convalescent period extended the average epidemic by 540 days (95% CI: 508–572 days), doubling the current length, despite an insignificant rise in the number of new cases generated.

**Conclusions/Significance:**

Our results show that reductions in the per sex act transmission probability via abstinence and condom use should reduce the number of sporadic sexual transmission events, but will not significantly reduce the epidemic size and may only minimally shorten the length of time the public health community must maintain response preparedness. While the number of infectious survivors is expected to greatly decline over the coming months, our results show that transmission events may still be expected for quite some time as each event results in a new potential cluster of non-sexual transmission. Precise measurement of the convalescent period is thus important for planning ongoing surveillance efforts.

## Author Summary

Researchers have recently raised suspicion that the Ebola virus can be transmitted sexually from survivors after recovering from the life-threatening acute phase characteristic of Ebola virus disease (EVD). However, the nature of the impact sexual transmission from convalescent survivors may have on disease dynamics remains unknown. Mathematical models are useful for translating empirical uncertainty into a range of possible outcomes. We formalized an epidemiological model that accounts for a secondary route of transmission of EVD through sexual contact with otherwise healthy survivors. We found that while very few additional cases are expected, a 3-month period of convalescent infectivity could extend the 2014–2015 Sierra Leone epidemic by nearly 3 months, and a 6-month convalescent period could double the current length by extending it an additional 18 months. Our results reveal that measures to reduce sexual contact between survivors and susceptible individuals are not likely to have a major impact on the length of time affected public health communities must remain vigilant, and highlight the need for ongoing surveillance efforts.

## Introduction

Recent reports suggesting the potential for sexual transmission of Ebola virus from convalescent survivors have raised a number of important questions regarding its impact on the final phase of the epidemic in West Africa [1,2]. Even once the worst hit countries of Guinea, Liberia, and Sierra Leone are declared free of Ebola virus disease (EVD), rare cases may still arise from the large number of remaining survivors. Importantly, sexual transmission is dependent on the frequency of infections rather than the density of available hosts, allowing chains of transmission to persist at low susceptible densities where non-sexual transmission would typically fail to occur [3]. Perhaps the most crucial element for bringing the epidemic to an end is maintaining vigilance in the community by preventing—or quickly responding to—new chains of transmission. Thus, it is important to investigate the potential impact of convalescent sexual transmission on the transmission dynamics in general, and on the tail of the epidemic in particular, to understand how long that vigilance might remain critical.

Follow-up studies on survivors of the 1995 outbreak in the Democratic Republic of Congo [4] and the 2000 [5] and 2007 [6] outbreaks in Uganda have raised awareness of what is now being termed “post-Ebola syndrome” (post-Ebola sequelae)–debilitating illnesses from myalgia to uveitis—which can persist for at least 21 months after the onset of symptoms. Though the virus is no longer detected in the blood after acute EVD symptoms disappear, active (replicating) virus has been documented in ocular fluid, rectal fluids, vaginal fluids, and semen [1,4,7,8]. Transmission to sexual partners was never confirmed in earlier outbreaks, but was suspected to have occurred in at least one instance [4]. Similarly, cases of sexual transmission of other hemorrhagic fever infections, notably by the closely related Marburg virus, have been suspected in the past [9,10]. Studies from the West African outbreak, showing viremia in semen 4–6 months after onset of symptoms in 65% of men tested (7–9 months in 26%) [1] and presenting molecular evidence of sexual transmission from a survivor 179 days after onset of symptoms [2], suggest that sexual transmission from convalescent men can and does occur.

Sexual transmission of Ebola virus from convalescent survivors is likely a rare event, but researchers have warned that it should be considered in epidemiological models that are used to predict the trajectory of an outbreak [11]. Without aiming to make a predictive model but rather to understand what aspects of the epidemic may be affected by inclusion of sexual transmission, we devised a novel formulation of the mathematical model for EVD transmission: SEICR (susceptible-exposed-infectious-convalescent-recovered), which includes a component for convalescent sexual transmission from convalescent survivors who maintain active Ebola virus replication. We illustrated the model by fitting it to weekly EVD incidence from Sierra Leone, the largest population of recovering survivors from the current West Africa epidemic. We performed sensitivity analysis to understand the influence of key unknown parameters, such as the duration of the convalescent period and the transmission probability per sexual contact. Considering the stochastic nature of such rare sexual transmission events, we also performed Monte Carlo simulations to explore the impact of sexual transmission on the epidemic tail in Sierra Leone.

## Methods

### Transmission model

**Fig 1:**
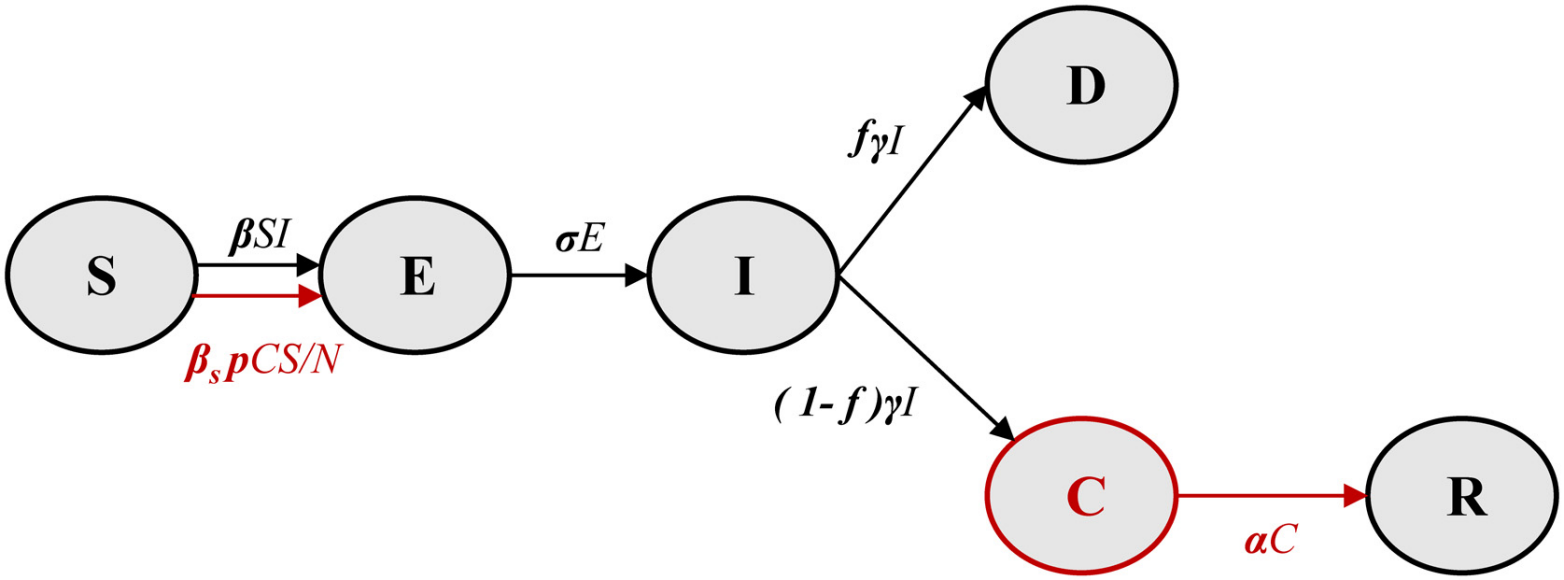
Schematic illustration of EVD transmission model including sexual transmission from convalescent patients. The elements in black form the base model without sexual transmission [12–14]. The red elements (convalescent individuals and additional transmission term) were added to account for sexual transmission.

We extended a SEIR (susceptible-exposed-infected-recovered) modeling framework, which has been extensively used to describe EVD transmission [12–14], by adding a component for sexual transmission from convalescent survivors who maintain active Ebola virus replication (Fig 1). The resulting SEICR model has five states: susceptible, *S*, exposed, *E*, symptomatic and infectious, *I*, convalescent, *C*, fully recovered and immune, *R*, and dead, *D*. The model is represented by the following set of ordinary differential equations (ODEs):

$$\begin{array}{l} \frac{dS}{dt}\;=\;\beta \;(t)\;S\;I-\beta_{S}\;p\;C\;\frac{S}{N}\\ \frac{dE}{dt}\;=\;\beta \;(t)\;S\;I+\beta_{S}\;p\;C\;\frac{S}{N}\;-\;\sigma E\\ \frac{dI}{dt}\;=\;\sigma E\;-\;\gamma \;I\\ \frac{dC}{dt}\;=\;(1-f)\;\gamma \;I\;-\;\alpha \;C\\ \frac{dR}{dt}\;=\;\alpha \;C\\ \frac{dD}{dt}\;=\;f\;\gamma \;I \end{array}$$ (1)

where *N = S + E + I + C + R* denotes the total population size. We assumed the non-sexual transmission rate, *β*(*t*), to be initially constant (*β*_0_) before it starts to decay exponentially due to the effect of control interventions and behavior change after time*τ*: *β*(*t*) = *β*_1_ + (*β*_0_ –*β*_1_)*e*^-*k*(*t*-*τ*)^ [12]. The sexual transmission parameter, *β*_s_, can be described as the product of two parameters (*β*_s_ = *ηq*) that we will consider separately: *η* is the per sex act transmission probability of Ebola virus from convalescent men, and *q* is the daily rate at which they engage in sexual intercourse. The number of convalescent individuals are scaled by *p*, which is the proportion of convalescent survivors who are sexually active men. 1/*σ* and 1/*γ* represent the average durations of incubation and symptomatic infection, respectively. *f* is the case fatality rate. The average duration after which convalescent patients recover completely and shed no further replicating Ebola virus from their body is given by 1/*α*. We assumed that sexual transmission is frequency-dependent [3,15,16], i.e., the probability that the sexual partner of a convalescent man is susceptible is given by *S*/*N*.

The basic reproductive number, *R*_0_, for the SEICR model can be calculated using the next-generation matrix method [17,18] and is given by

$$\;R_{0}=\frac{\beta S_{0}}{\gamma }+\frac{\left(1-f\right)\;p\;\beta_{s}\;}{\alpha },$$

where *S*_0_ is the initial number of susceptible individuals (see S1 Appendix). When *α* goes to infinity or either *β*_s_ = 0 or *p* = 0, the equation reduces to the basic reproductive number in absence of sexual transmission: $R_{0,N}=\frac{\beta S_{0}}{\gamma }$. Thus, the second term represents the contribution of sexual transmission from convalescent patients to the overall *R*_0_: $R_{0,C}=\frac{\left(1-f\right)\;p\;\beta_{s}}{\alpha }\;$.

### Model parameters

Since the number of sexual transmission events was likely to be small and little information is currently available on the nature of each transmission event, we fitted only the non-sexual (SEIR) deterministic EVD transmission model to weekly incidence of confirmed and probable cases in Sierra Leone as reported in the WHO patient database [19] (S1 Fig). The data set was extended with weekly incidence from the situation report for the most recent weeks when no data was available in the patient database. In order to account for variability in the accuracy of reporting, we assumed that the number of reported cases follows a negative binomial distribution with mean as predicted by the model and dispersion parameter *ϕ* [20]. We derived maximum likelihood estimates (MLE) of the following model parameters [14,21]: the baseline transmission rate *β*_0_, the time *τ* at which transmission starts to drop, the rate *k* at which transmission decays, and the dispersion parameter *ϕ*. For the fitting procedure, we assumed that there were no sexual transmission events, i.e., we set *β*_S_ to zero. The basic reproductive number in absence of sexual transmission is *R*_0,*N*_ = *β*_0_*N*_0_/*γ*, and the reproductive number in presence of partially effective control interventions is *R*_1,*N*_ = *β*_1_*N*_0_/*γ*, with *N*_0_ being the population size of Sierra Leone. We explored value ranges for sexual transmission parameters (Table 1) based on information from the current epidemic [22] and studies of human immunodeficiency virus [23,24]. Remaining parameters were based on published values from the literature (Table 1).

**Table 1:** Model parameters describing EVD transmission in Sierra Leone. The indicated parameter ranges are used for the sensitivity analysis (partial rank correlation coefficients, PRCC) only.

Parameter		Value (Range)	Comments and References
Basic reproductive number without sexual transmission	*R*_0,*N*_	2.13 (1.26–2.53)	Estimated—Range explores estimates from [13,25]
Basic reproductive number in presence of control interventions	*R*_1,*N*_	0.67	Estimated
Date of onset of symptoms in index case		April 23, 2014	[13,25,26]
Rate at which transmission rate decays	k	0.011 d^-1^	Estimated
Time at which transmission rate starts to decay	τ	51.0 d	Estimated
Incubation period	1/σ	11.4 d	[22]
Infectious period	1/γ	3.9 d	Together with the incubation period results in a generation time of 15.3 d [22]
Dispersion parameter	ϕ	0.050	Estimated
Initial population size	*N*_0_	6.316x10^6^	Based on 2014 estimate [27]
Sexual transmission probability (per coital act)	*η*	0.001 (0.0005–0.002)	Roughly based on sexual transmission probability of HIV per coital act from infected men [24]
Case fatality rate	*f*	0.69	[22]
Frequency of sex acts	*q*	0.272 d^-1^ (0.136–0.544)	8.27 coital acts per month [23]
Proportion of convalescent survivors who are infectious and sexually active	*p*	0.347 (0.1725–0.694)	Of 47.4% male survivors, 73.1% are aged 15–45 [22]
Rate at which convalescent survivors recover completely	*α*	1/87.35 d^-1^ (1/174.7–1/43.7)	3 months after onset of symptoms [4,28] and assuming an infectious period of 3.9 days

### Deterministic model and sensitivity analysis

We solved the system of ODEs numerically using the function ‘*ode’* from the ‘*deSolve’* package in the R software environment for statistical computing [29]. We compared the following response variables of the model: the epidemic peak number of exposed, *E*, acute, *I*, and convalescents, *C*, cases; the cumulative number of EVD cases, deaths, and recoveries; the date at the epidemic peak; the daily and cumulative incidence of sexual transmission; and the date at which the last symptomatic case either died or entered into convalescence (“day of last case”). We defined the day of last case as the time when the number of symptomatic and infectious individuals, *I*, dropped below 0.5. We considered the following parameters for the sensitivity analysis: the per sex act transmission probability of Ebola virus from convalescent men (*η*), the proportion of convalescent survivors who are sexually active men (*p*), the rate at which they engage in sexual intercourse (*q*), and the rate at which convalescent patients recover completely and shed no further replicating Ebola virus from their body (*α*). The sensitivity of the response variables to changes in *R*_0_ was explored simultaneously as a comparison. We generated 1000 parameter combinations from the uniform ranges, log-transformed [0.5x – 2x] for the parameter values for *η*, *p*, *q*, and *α*, given in Table 1 via Latin hypercube sampling using the Huntington and Lyrintzis correlation correction method (function ‘*lhs*’ from R package ‘*pse*’) [30]. We then calculated partial rank correlation coefficients (PRCCs) using 50 bootstrap replicates [31].

### Monte Carlo simulations

We performed stochastic simulations of the SEICR model with and without sexual transmission using Gillespie’s algorithm [32]. We specifically investigated the following response variables from the simulations: the cumulative number of EVD cases, the size and date of the epidemic’s peak incidence (daily number of new symptomatic infections), and the date of last case (last day that symptomatic infections, *I*, fell below 1). Summary statistics were based on the results of 1000 simulation runs for each transmission scenario. We calculated the average of the peak and total cumulative number of EVD cases by including all simulations runs, i.e., also the simulations that rapidly go extinct. In contrast, the average dates of the epidemic peak and last case were based on the simulated epidemic trajectories over which 50 or more cases were accumulated.

## Results

### Contribution of sexual transmission to overall R_0_

Assuming a conservative baseline scenario (*η* = 0.001 and 1/ *α* = 3 months; Table 1), the reproductive number of a convalescent infection, *R*_0,*C*_, is 0.0024. This corresponds to only 0.12% of the overall *R*_0_ of 2.0224. Increasing the convalescent period from 3 to 6 months, the contribution of *R*_0,C_ (0.0051) to the overall *R*_0_ rises to 0.25%. The equation for *R*_0,C_ (see Methods) illustrates that doubling the per sex act transmission probability has the same impact as doubling the convalescent period. It is important to note that the relative contribution of sexual transmission to the overall reproductive number rises as the effective reproductive number drops during the epidemic due to the effects of control interventions and decreasing density of susceptible hosts (see S2 Fig).

### Effect of sexual transmission parameters on epidemic dynamics

**Fig 2:**
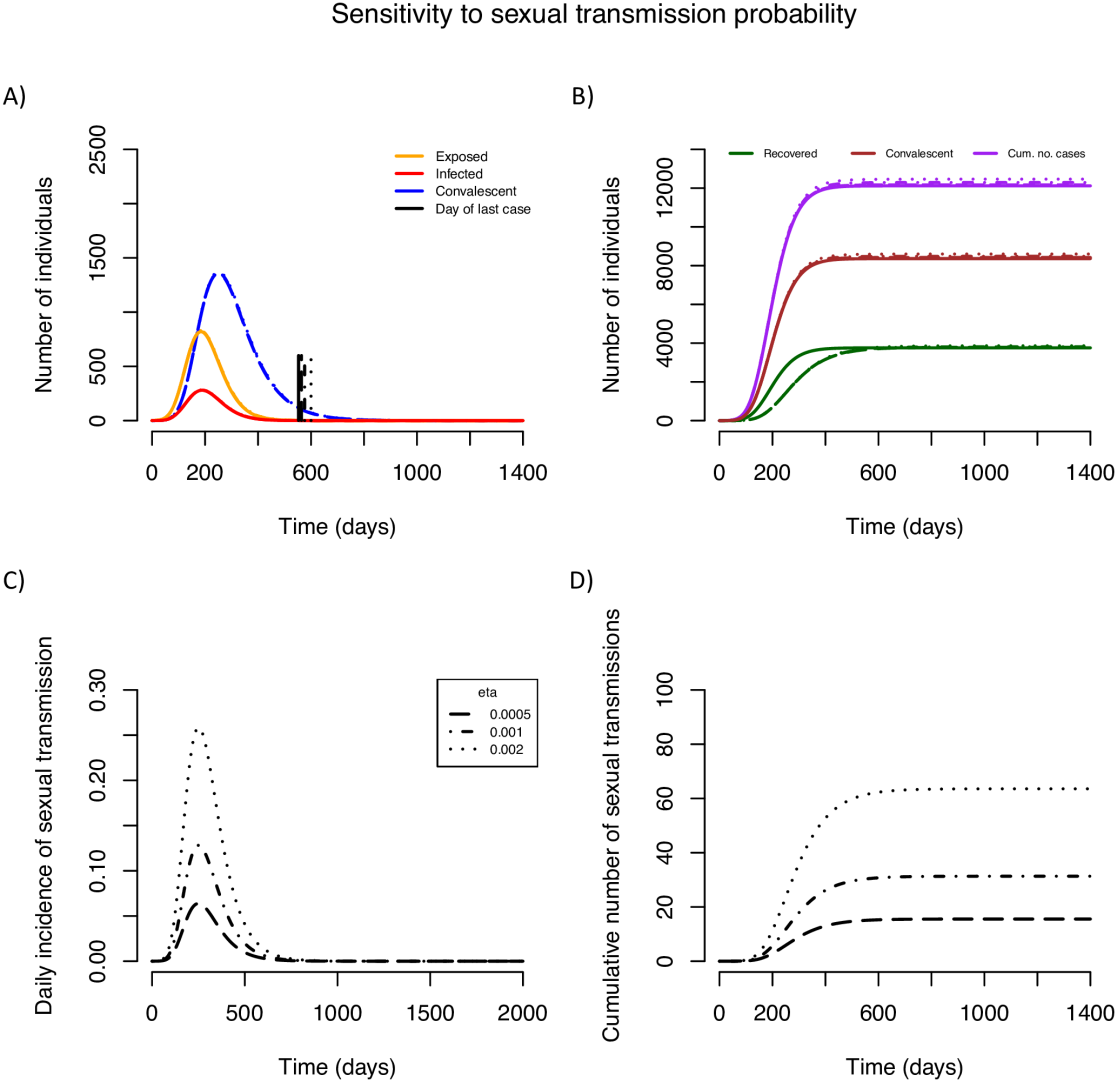
Effect of convalescent period on EVD epidemics. The average duration of the convalescent period (1/*α*) is varied between 3 and 9 months. (A, B): Epidemic trajectories in presence (broken lines) and absence of sexual transmission (solid lines). Vertical lines mark the day of last symptomatic case. (C) Daily incidence of sexual transmission. (D) Cumulative number of sexual transmission events. Note that the vertical axes vary across panels.

**Fig 3:**
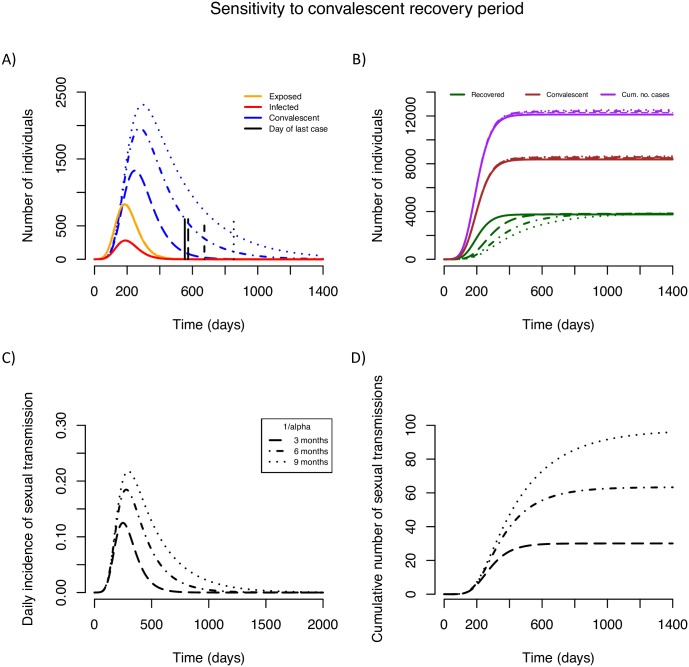
Effect of per sex act transmission probability on EVD epidemics. The per sex act transmission probability (*η*) is varied between 0.05% and 0.2%. (A, B): Epidemic trajectories in presence (broken lines) and absence of sexual transmission (solid lines). Vertical lines mark the day of last symptomatic case. (C) Daily incidence of sexual transmission. (D) Cumulative number of sexual transmission events. Note that the vertical axes vary across panels.

**Fig 4:**
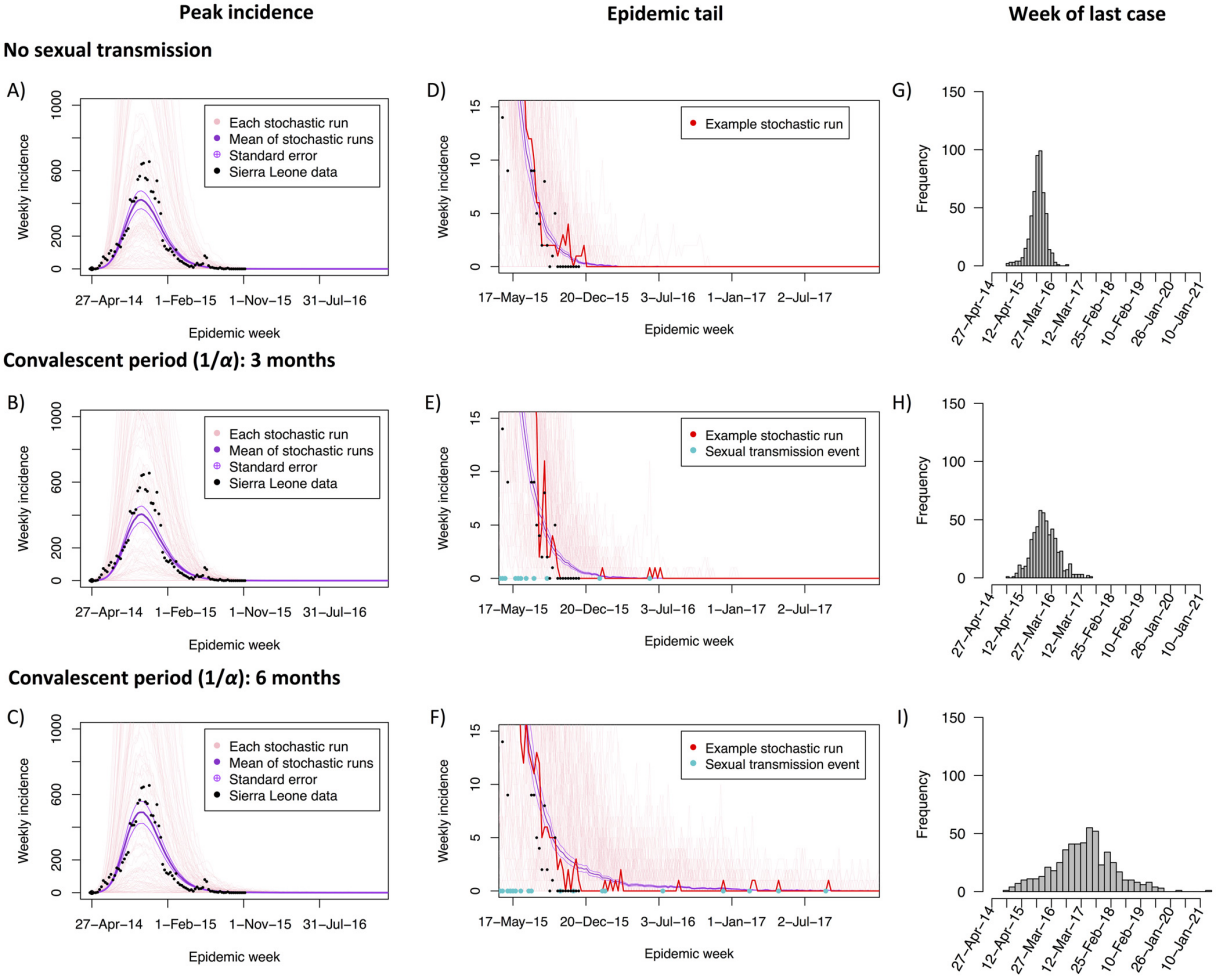
Impact of convalescent period on the tail of the EVD epidemic. Monte Carlo simulations of the weekly incidence of new cases (A, B, C) and the sporadic occurrence of sexual transmission events at the epidemic tail (D, E, F), assuming no sexual transmission (A, D), and sexual transmission with a 3 (B, E) and 6 months (C, F) convalescent period and a per sex act transmission probability fixed at 0.001. Light red lines show the result of 200 simulated trajectories, with corresponding mean (thick purple line) and standard errors (thin purple lines). Black dots denote incident cases in Sierra Leone as reported by the WHO [19]. The dark red lines in (D), (E), and (F) highlight a single representative trajectory, and the blue dots along the horizontal axis indicate a sexual transmission event. Histograms of the day of the last EVD case from 1,000 simulated epidemics in absence of sexual transmission (G) and for a convalescent period of 3 (H) and 6 (I) months.

The two key unknown parameters of sexual transmission are the per sex act transmission probability, *η*, and the rate at which convalescent survivors fully recover, *α*. Both parameters were found to have very small effects on the peak number of infected or exposed patients (Figs 2A, 3A, 4A, 4B and 4C; S2 and S3 Figs). The duration of the convalescent period has a large impact on the peak number of convalescent individuals, while *η* does not (compare Fig 2A and Fig 3A). The total number of recovered individuals is reached more slowly the longer the convalescent period (Fig 2B), which is not an effect caused by *η* (Fig 3b). While the convalescent periods (1/ *α* = [3–9 months]) and the values of *η* (*η* = [0.0005–0.002]) we explored create very few extra cases (Figs 2B and 3B), sensitivity analyses revealed that a higher per sex act transmission probability, *η*, a higher proportion of sexually active convalescent individuals, *p*, or a higher frequency of sex acts, *q*, have larger impacts on the total number of cases than would proportional increases in the convalescent period (see S3A Fig). Sensitivity analyses also revealed that these sexual transmission parameters could produce a small delay in the epidemic peak, more so than would changes in the convalescent period (see S3B Fig).

The number of sexual transmission events expected from the baseline scenario (*η* = 0.001 and 1/ *α* = 3 months) is 31.2, the majority of which will occur around the peak of the epidemic (Figs 2C and 3D) and thus likely go undetected. Doubling either *η* or 1/ *α* results in nearly equal increases in the incidence and cumulative number of sexual transmission events (Figs 2C, 2D, 3C and 3D), with either leading to roughly double the number of sexually transmitted cases over the course of the whole epidemic (> 60 cases). It should be noted that the total number of cases increases more than by simply the number of sexual transmission events, because each sexual transmission event results in a new potential cluster of non-sexual transmission. The day of last case is affected more by the convalescent period than the per sex act transmission probability (represented by vertical lines in Figs 2A and 3A), a result confirmed by the sensitivity analysis (see S3A Fig). The tail of the epidemic will depend on a small number of events that are likely to be affected by stochastic processes, thus we used Monte Carlo simulations to explore this behaviour.

### Impact of sexual transmission on the epidemic tail

We performed stochastic simulations of the EVD transmission model to investigate the epidemic dynamics when the number of new cases becomes small, i.e, during the tail of the epidemic. Comparing model simulations while assuming a convalescent period of 3 months to those without sexual transmission confirmed the deterministic results that sexual transmission from convalescent survivors does not lead to a significant increase in the cumulative number of infected cases (non-STI: 11,092 +/- 627 cases; STI: 10,944 +/- 642 cases; Wilcox rank sum test: W = 491990, p = 0.53), nor the size (non-STI: 77 +/- 4.1 new cases per day; STI: 75 +/- 4.2 new cases per day; W = 493710, p = 0.62) or timing (non-STI: day 187 +/- 0.9; STI: day 187 +/- 0.9; t = 0.19, df = 1017.4, p = 0.85) of the epidemic peak incidence (Fig 4A, 4B and 4C). This conservative period of potential sexual transmission, which has recently been shown to extend well beyond 3 months in at least 65% of patients [1], lengthened the average date on which the last active case could be detected by nearly three months (non-STI: 548 +/- 4.0 days; STI: 630 +/- 6.6 days; difference: 83 days [95% CI: 68–98 days], *t* = -10.8, df = 867.97, p < 0.0001; Fig 4D, 4E, 4G and 4H). The width of the tail (s.d. = 151 days) was such that 23.4% of the 529 simulated epidemics that accumulated at least 50 cases still experienced symptomatic individuals 730 days (two years) after the start of the epidemic (Fig 4H). Strikingly, when the convalescent period was extended from 3 months to 6 months, the projected length of the epidemic increased to a mean of 1088 days (+/- 15.5), with 84.0% of the 538 sustained epidemics taking over two years to end (Fig 4F and 4I). However, the average number of new cases produced remained small (11,869 +/- 663 cases; W = 482790, p = 0.18). Importantly, there is greater variance in the tail of the epidemic when sexual transmission is considered, and this uncertainty grows with the length of the convalescent period (Fig 4G, 4H and 4I).

To understand the differential impacts of the convalescent period (1/ *α*) and the per sex act transmission probability (*η*) on the epidemic tail, the effect of two-fold reductions in *η* on the average duration of the epidemic under both 3 and 6 month convalescent periods were compared (S1 Table). Cutting the per sex act transmission probability in half led to statistically significant reductions in the length of the epidemic, but this was 3-fold less effective than equivalent changes in the convalescent period (S4 Fig and S1 Table), corroborating the results of the deterministic sensitivity analyses (above). We also note that reducing *η* did not greatly reduce the enormous variance observed with the longer convalescent period.

## Discussion

Our study shows that the length of the convalescent period will determine whether or not sexual transmission of Ebola virus from recovering patients will have a profound effect on the length of time it will take for the epidemic to completely fade. For Sierra Leone, we found that an average convalescent period of 3 months, and a per sex act transmission probability of 0.1%, could extend the EVD epidemic in Sierra Leone by an average of 83 days (95% CI: 68–98 days). Such a scenario would be consistent with the occurrence of a small number of sexual transmission events during the end-phase of the epidemic. However, assuming an average convalescent period of 6 months led to simulated epidemics whose tails were much more variable, and much longer, despite a lack of significant increase in the total number of cases. So far, the reported cases of sexual transmission of EVD remain rare [1,2]. Hence, the per sex act transmission probability of Ebola virus from male convalescent survivors is unlikely to be higher than 0.1%, and might well be below this value. Our sensitivity analysis indicates that the duration of the EVD epidemic is heavily influenced by the period during which convalescent men can transmit sexually, calling for a better understanding of the persistence and duration of infectivity of Ebola virus RNA in convalescent patients.

We extended an accepted modeling framework that has been widely used to describe the epidemic trajectories of EVD outbreaks and epidemics [12–14]. To our knowledge, this is the first study using mathematical modeling to assess the potential impact of sexual transmission of Ebola virus on the epidemic in West Africa. In addition, given the generality of the model, this is also the first model that investigates the impact of including a secondary transmission route from convalescent individuals. Similar compartmental models have been formalized to account for more realistic infectious periods, including both infectious relapse [33,34] and progression through classes of varying stages of infectivity [35,36]. However, none of these models included a change in transmission mode between the primary and subsequent infectious classes. This model, then, may also have implications for other pathogens with this kind of secondary transmission route (e.g. some adenoviruses [37]; see [38] for examples across mammal species) including other neglected tropical diseases, such as African sleeping sickness [39], other hemorrhagic fevers that display pathologies similar to EVD [9,10], and the most recent emergence of Zika virus [40,41].

In the absence of a better understanding of sexual transmission of EVD, mathematical modeling currently remains the only tool to explore its potential impact on the epidemic trajectory. There are several extensions to this work that future models should consider including in order to make accurate predictions. These include: transmission by other bodily fluids (e.g., vaginal secretions [4,7]), asymptomatic infection [42,43], considering spatial aspects of both social and sexual transmission [44], and heterogeneity in sexual behavior [3,45]. Sexual behaviors, for instance, are often specific to a given culture, and may change drastically in response to such a devastating epidemic that can destroy entire communities and create stigma, disrupting existing social and sexual contact networks [46,47]; the lack of these details particularly in developing countries presents a challenge for parameterizing a more complex model. We assumed the duration of convalescence to be exponentially distributed. Eggo *et al*. [48] fitted a series of unimodal distributions to the data on Ebola virus RNA detection in semen recently reported by Deen *et al*. [1] and found that the convalescence period could be best described by a gamma distribution. Furthermore, Deen *et al*. [1] found that the cycle-threshold values decreased over time, indicating that the Ebola virus load in semen and the viral infectivity might also decrease during the convalescence period. It is also critical to measure the length of time viral particles persisting in the seminal fluid remain infectious. Molecular techniques to detect intermediate (replicative) positive-sense RNA stages of the virus, infection of human cell lines in tissue culture, or tests in animal models are some typical methods. Retrospective studies using phylodynamics could also prove helpful for estimating this type of parameter [25,49]. Uncertainty in the data is not limited to sexual transmission; we fitted our model to weekly incidence of confirmed and probable cases in Sierra Leone, but did not take into account potential underreporting as others have done recently [36]. In addition, the incidence data to which we fit our model will, for the most part, be driven by direct transmission of the virus and thus, to better parameterize and estimate the risk of sexual transmission, we would need data with greater resolution (e.g. knowledge of which cases were caused by sexual transmission events). Another caveat is that EVD is known to exhibit superspreading characteristics [50,51], and superspreading events could lead to explosive regrowth of the epidemic after the occurrence of a new case through sexual transmission [50]. And finally, like other negative-sense single-stranded RNA ((-)ssRNA) viruses [52], the species currently circulating in West Africa has been estimated to have high substitution rates [26,53,54]. This rapid evolution detected throughout the current outbreak zone suggests that within- or between-host adaptation of the virus leading to pro-longed persistence in the seminal fluids is possible. However, without significant attenuation of EVD’s high mortality and morbidity virulence, evolution of sexual transmission becoming the primary route of spread is highly unlikely, as the subsequent infections that arise from a sexual transmission event will be caused primarily through transmission during the acute non-sexual transmission phase of the infection.

Awareness of the potential for sexual transmission has led to WHO issuing recommendations that ask convalescent men to abstain from sexual activity as much as possible and to use condoms for up to 6 months after the onset of symptoms [28]. Condom use and social awareness of the risks of sexual transmission during convalescence should reduce the per sex act transmission probability (*η*) and the frequency of sex acts (*q*), respectively. Our results suggest that while such interventions should reduce the number of sporadic sexual transmission events, they will not necessarily reduce the overall number of cases nor the length of time during which the public health community must stay vigilant in responding to these sporadic cases because they will not affect the time during which convalescent survivors can shed infectious virus (1/*α*). This is especially poignant since adherence to these recommendations will never be 100%, particularly after the threat from symptomatic individuals passes. Thus, our results suggest that the current requirement for declaring a region free from EVD (42 days following either death or a second negative RT-PCR test of the blood from the last known patient), may be too short. Sierra Leone was first declared free from EVD on 7 November 2015 [55], but the case of a young woman who died from Ebola in January [56] highlights the need for the 90-day period of enhanced surveillance after the declaration has been made. The WHO report that 10 such “flare-ups”, or cases with no apparent link to the original acute symptomatic transmission chains, have been identified throughout the region, and are suspected to have resulted from contact with infectious survivors [57]. However, none of these events have caused a major resurgence of new cases. This is likely primarily due to the continuing vigilance, awareness, and resources provided by public health infrastructures, and is reflected by the basic reproduction number in presence of control interventions, *R*_1,*N*_ < 1 (Table 1). A relaxation of current response and surveillance efforts could allow a rare sexual transmission event to propagate a new epidemic.

As more data about the convalescent survivors of EVD becomes available, this and future mathematical modeling studies will help to better understand the potential epidemiological consequences of sexual transmission on EVD epidemics. Precise estimates of key parameters are important for providing convalescent survivors with sound advice that balances protection of the community with the harm that could come from unnecessary stigmatization [46,58,59].

## Supporting Information

S1 Fig Dynamics of Ebola virus disease (EVD) epidemic in Sierra Leone. Model fit to weekly incidence of confirmed and probable cases are shown together with data from the patient database as reported by WHO (circles) [19]. The shaded area corresponds to the 95% prediction interval, assuming that the number of reported cases follows a negative binomial distribution.
(PDF)

S2 Fig Contribution of sexual transmission to the overall reproductive number. The relative contribution of *R*_0,*C*_ to the overall reproductive number increases over the course of the epidemic, as the non-sexual transmission rate falls due to the implementation of control measures and as the number of susceptible individuals declines. The dashed line indicates the day control measures were implemented (τ = 51 days after the index case).
(PDF)

S3 Fig Results from the deterministic sensitivity analysis. Partial rank correlation coefficients (PRCCs) and 95% confidence intervals for *η* (per sex act transmission probability), *p* (frequency of sex acts), *q* (proportion of the convalescent individuals who are male and sexually active), and 1/*α* (convalescent period), as well as the reproductive number *R*_0_. (A) Sensitivity of the duration and size of the epidemic on changes of parameters. (B) Sensitivity of the timing and daily incidence of symptomatic cases, *I*, at the peak of the epidemic on changes of parameters.
(PDF)

S4 Fig Sexual transmission parameter value effects on epidemic length. The effects of two-fold changes in convalescent period (1/ *α*) and in per sex act sexual transmission probability (*η*) on the average duration of Monte Carlo simulated Ebola virus epidemics. Box and whisker plots show the mean and variance for the length of those epidemics (number of days symptomatic cases remained in the population) which sustained 50 or more total cases (out of 1000 simulations). Statistical significance of comparisons were corrected for multiple tests following the Games-Howell method, and all are reported in *Supporting Information* S1 Table. Asterisks denote p-values of (*) < 0.05 and (***) <0.0001.
(PDF)

S1 Table Sexual transmission parameter value effects on epidemic length. Pairwise effects of two-fold changes in convalescent period (1/ *α*) and in per sex act sexual transmission probability (*η*) on the average duration of Monte Carlo simulated Ebola virus epidemics. Out of 1000 simulations for each set of parameters (*α* and *η*), the mean length (number of days) of the epidemics having at least 50 total cases (n = number of simulations) is given in the diagonal. Statistical significance of each pairwise comparison is given above the diagonal (t statistic (df) and p-value) and were corrected for multiple tests using the Games-Howell method. The relative reduction in the length of the epidemic (% fewer days) is given below the diagonal. The red and blue values are those referenced in the results of the manuscript.
(PDF)

S1 Appendix Calculating *R*_0_. (PDF)

S2 Appendix R codes and data file for modeling sexual transmission of Ebola virus disease. *Sierra Leone incidence data*. As reported by the World Health Organization on their Ebola outbreak data web page. *Parameter Estimation (SEIR)*. *Implementing deterministic EVD models (SEIR*, *SEICR)*. *Implementing sensitivity analysis*. *Implementing Monte Carlo simulations of EVD models*. *Summary statistics of Monte Carlo simulations*.
(ZIP)

## References

[1] Deen GF, Knust B, Broutet N, Sesay FR, Formenty P, Ross C, et al Ebola RNA Persistence in Semen of Ebola Virus Disease Survivors—Preliminary Report. N Engl J Med. 2015 10.1056/NEJMoa1511410 PMC5798881 26465681

[2] Mate SE, Kugelman JR, Nyenswah TG, Ladner JT, Wiley MR, Cordier-Lassalle T, et al Molecular Evidence of Sexual Transmission of Ebola Virus. N Engl J Med. 2015 10.1056/NEJMoa1509773 PMC4711355 26465384

[3] Anderson RM, May RM. Infectious Diseases of Humans: Dynamics and Control. Oxford: Oxford Univeristy Press; 1991.

[4] Rowe AK, Bertolli J, Khan AS, Mukunu R, Muyembe-Tamfum JJ, Bressler D, et al Clinical, Virologic, and Immunologic Follow-Up of Convalescent Ebola Hemorrhagic Fever Patients and Their Household Contacts, Kikwit, Democratic Republic of the Congo. J Infect Dis. 1999;179 Suppl: 28–35. 10.1086/514318 9988162

[5] Clark D V, Kibuuka H, Millard M, Wakabi S, Lukwago L, Taylor A, et al Long-term sequelae after Ebola virus disease in Bundibugyo, Uganda: a retrospective cohort study. Lancet Infect Dis. Elsevier Ltd; 2015;3099: 1–8. 10.1016/S1473-3099(15)70152-0 25910637

[6] Bausch DG, Towner JS, Dowell SF, Kaducu F, Lukwiya M, Sanchez A, et al Assessment of the risk of Ebola virus transmission from bodily fluids and fomites. J Infect Dis. 2007;196 Suppl: S142–S147. 17940942 10.1086/520545

[7] Rodriguez LL, De Roo A, Guimard Y, Trappier SG, Sanchez A, Bressler D, et al Persistence and genetic stability of Ebola virus during the outbreak in Kikwit, Democratic Republic of the Congo, 1995. J Infect Dis. 1999;179 Suppl: S170–6. 9988181 10.1086/514291

[8] Varkey JB, Shantha JG, Crozier I, Kraft CS, Lyon GM, Mehta AK, et al Persistence of Ebola Virus in Ocular Fluid during Convalescence. N Engl J Med. 2015;372: 2423–2427. 10.1056/NEJMoa1500306 25950269 PMC4547451

[9] Ergonul O, Battal I. Potential sexual transmission of Crimean-Congo hemorrhagic fever infection. Jpn J Infect Dis. 2014;67: 137–8. 24647261 10.7883/yoken.67.137

[10] Slenczka W, Klenk HD. Forty Years of Marburg Virus. J Infect Dis. 2007;196: S131–135. 17940940 10.1086/520551

[11] Rogstad KE, Tunbridge A. Ebola virus as a sexually transmitted infection. Curr Opin Infect Dis. 2015;28: 83–85. 10.1097/QCO.0000000000000135 25501666

[12] Chowell G, Hengartner NW, Castillo-Chavez C, Fenimore PW, Hyman JM. The basic reproductive number of Ebola and the effects of public health measures: the cases of Congo and Uganda. J Theor Biol. 2004;229: 119–126. 15178190 10.1016/j.jtbi.2004.03.006

[13] Althaus CL. Estimating the Reproduction Number of Ebola Virus (EBOV) During the 2014 Outbreak in West Africa. PLOS Curr Outbreaks. 2014; 1–9. 10.1371/currents.outbreaks.91afb5e0f279e7f29e7056095255b288 PMC4169395 25642364

[14] Althaus CL, Low N, Musa EO, Shuaib F, Gsteiger S. Ebola virus disease outbreak in Nigeria: Transmission dynamics and rapid control. Epidemics. Elsevier B.V.; 2015;11: 80–84. 10.1016/j.epidem.2015.03.001 25979285

[15] Thrall PH, Antonovics J. Polymorphism in sexual versus non-sexual disease transmission. Proc R Soc B Biol Sci. 1997;264: 581–587.

[16] Garnett GP. An introduction to mathematical models in sexually transmitted disease epidemiology. Sex Transm Infect. 2002;78: 7–12. 11872850 10.1136/sti.78.1.7 PMC1763694

[17] Diekmann O, Heesterbeek JAP. Mathematical Epidemiology of Infectious Diseases. Chichester: John Wiley and Sons; 2000.

[18] Diekmann O, Heesterbeek JAP, Metz JAJ. On the definition and the computation of the basic reproduction ratio R0 in models for infectious diseases in heterogeneous populations. J Math Biol. 1990;28: 365–382. 2117040 10.1007/BF00178324

[19] World Health Organization. Ebola data and statistics: Data on new cases per epi week for Sierra Leone [Internet]. 2015 [cited 18 Nov 2015]. Available: http://apps.who.int/gho/data/node.ebola-sitrep.ebola-country-SLE-20151118?lang=en

[20] Kucharski AJ, Camacho A, Flasche S, Glover RE, Edmunds WJ, Funk S. Measuring the impact of Ebola control measures in Sierra Leone. Proc Natl Acad Sci. 2015;112: 201508814. 10.1073/pnas.1508814112 PMC4655521 26460023

[21] Althaus CL. Rapid drop in the reproduction number during the Ebola outbreak in the Democratic Republic of Congo PrePrints. PeerJ Prepr. 2015;3: e1418. 10.7717/peerj.1418 PMC4655090 26618087

[22] WHO Ebola Response Team. Ebola Virus Disease in West Africa—The First 9 Months of the Epidemic and Forward Projections. Engl New J Med. 2014;371: 1481–1495. 10.1056/NEJMoa1411100 PMC4235004 25244186

[23] Gray RH, Wawer MJ, Brookmeyer R, Sewankambo NK, Serwadda D, Wabwire-Mangen F, et al Probability of HIV-1 transmission per coital act in monogamous, heterosexual, HIV-1-discordant couples in Rakai, Uganda. Lancet. 2001;357: 1149–1153. 11323041 10.1016/S0140-6736(00)04331-2

[24] Wawer MJ, Gray RH, Sewankambo NK, Serwadda D, Li X, Laeyendecker O, et al Rates of HIV-1 transmission per coital act, by stage of HIV-1 infection, in Rakai, Uganda. J Infect Dis. 2005;191: 1403–1409. 15809897 10.1086/429411

[25] Alizon S, Lion S, Murall CL, Abbate JL. Quantifying the epidemic spread of Ebola virus (EBOV) in Sierra Leone using phylodynamics. Virulence. 2015;5: 825–827. 10.4161/21505594.2014.976514 PMC4601477 25495064

[26] Gire SK, Goba A, Andersen KG, Sealfon RSG, PD J, Kanneh L, et al Genomic surveillance elucidates Ebola virus origin and transmission during the 2014 outbreak. Science (80-). 2014;345: 1369–1372. 10.1126/science.1259657 PMC4431643 25214632

[27] The World Bank. Sierra Leone country at a glance [Internet]. 2015. Available: http://www.worldbank.org/en/country/sierraleone

[28] World Health Organization. WHO website [Internet]. Available: http://www.who.int/reproductivehealth/topics/rtis/ebola-virus-semen/en/

[29] R Core Team. R: A Language and Environment for Statistical Computing. Vienna, Austria: R Foundation for Statistical Computing; 2014.

[30] Chalom A, Prado PI. Parameter space exploration of ecological models. arXiv Prepr. 2012; arXiv:1210.6278.

[31] Blower SM, Dowlatabadi H. Sensitivity and Uncertainty Analysis of Complex Models of Disease Transmission: An HIV Model, as an Example. Int Stat Rev. 1994;62: 229–243.

[32] Gillespie DT. Exact Stochastic Simulation of couple chemical reactions. J Phys Chem. 1977;81: 2340–2361.

[33] Johnson TL, Landguth EL, Stone EF. Modeling Relapsing Disease Dynamics in a Host-Vector Community. PLoS Negl Trop Dis. 2016;10: e0004428 10.1371/journal.pntd.0004428 26910884 PMC4765964

[34] Schuette MC. A qualitative analysis of a model for the transmission of varicella-zoster virus. Math Biosci. 2003;182: 113–126. 12591619 10.1016/s0025-5564(02)00219-5

[35] Lloyd AL. Destabilization of epidemic models with the inclusion of realistic distributions of infectious periods. Proc Biol Sci. 2001;268: 985–93. 11370974 10.1098/rspb.2001.1599 PMC1088698

[36] Lloyd AL. Realistic Distributions of Infectious Periods in Epidemic Models: Changing Patterns of Persistence and Dynamics. Theor Popul Biol. 2001;60: 59–71. 11589638 10.1006/tpbi.2001.1525

[37] Swenson PD, Lowens MS, Celum CL, Hierholzer JC. Adenovirus types 2, 8, and 37 associated with genital infections in patients attending a sexually transmitted disease clinic. J Clin Microbiol. 1995;33: 2728–2731. 8567914 10.1128/jcm.33.10.2728-2731.1995 PMC228564

[38] Dejucq N, Jégou B. Viruses in the Mammalian Male Genital Tract and Their Effects on the Reproductive System Viruses in the Mammalian Male Genital Tract and Their Effects on the Reproductive System. 2001;65: 208–231. 10.1128/MMBR.65.2.208-231.2001 PMC99025 11381100

[39] Rocha G, Martins A, Gama G, Brandão F, Atouguia J. Possible cases of sexual and congenital transmission of sleeping sickness [5]. Lancet. 2004;363: 247. 10.1016/S0140-6736(03)15345-7 14738812

[40] Foy BD, Kobylinski KC, Chilson Foy JL, Blitvich BJ, Travassos da Rosa A, Haddow AD, et al Probable Non–Vector-borne Transmission of Zika Virus, Colorado, USA. Emerg Infect Dis. 2011;17: 880–882. 10.3201/eid1705.101939 21529401 PMC3321795

[41] Musso D, Roche C, Robin E, Nhan T, Teissier A, Cao-Lormeau VM. Potential sexual transmission of zika virus. Emerg Infect Dis. 2015;21: 359–361. 10.3201/eid2102.141363 25625872 PMC4313657

[42] Leroy EM, Baize S, Volchkov VE, Capron M, Debré P, Mccormick JB, et al Human asymptomatic Ebola infection and strong inflammatory response. Lancet. 2000;355: 2210–2215. 10881895 10.1016/s0140-6736(00)02405-3

[43] Bellan SE, Pulliam JRC, Dushoff J, Meyers LA. Ebola control: effect of asymptomatic infection and acquired immunity. Lancet. Elsevier Ltd; 2014;384: 1499–1500. 10.1016/S0140-6736(14)61839-0 PMC4829342 25390569

[44] Merler S, Ajelli M, Fumanelli L, Gomes MFC, Piontti APY, Rossi L, et al Spatiotemporal spread of the 2014 outbreak of Ebola virus disease in Liberia and the effectiveness of non-pharmaceutical interventions: a computational modelling analysis. Lancet Infect Dis. Elsevier Ltd; 2015;3099: 1–8. 10.1016/S1473-3099(14)71074-6 PMC4409131 25575618

[45] Smith J, Nyamukapa C, Gregson S, Lewis J, Magutshwa S, Schumacher C, et al The Distribution of Sex Acts and Condom Use within Partnerships in a Rural Sub-Saharan African Population. PLoS One. 2014;9: e88378 10.1371/journal.pone.0088378 24558387 PMC3928170

[46] Davtyan M, Brown B, Folayan MO. Addressing Ebola-related Stigma: Lessons Learned from HIV/AIDS. Glob Health Action. 2014;7: 1–4. 10.3402/gha.v7.26058 PMC4225220 25382685

[47] Van Bortel T, Basnayake A, Wurie F, Jambai M, Koroma S, Muana AT, et al Psychosocial effects of an Ebola outbreak at individual, community and international levels. Bull World Health Organ. 2016;94: 210–214. 10.2471/BLT.15.158543 26966332 PMC4773931

[48] Eggo RM, Watson CH, Camacho A, Kucharski AJ, Funk S, Edmunds WJ. Duration of Ebola virus RNA persistence in semen of survivors: population level estimates and projections. Eurosurveillance. 2015;20: pii = 30083 10.2807/1560-7917.ES.2015.20.48.30083 26676163

[49] Stadler T, Kühnert D, Rasmussen D a, Plessis L. Insights into the Early Epidemic Spread of Ebola in Sierra Leone Provided by Viral Sequence Data. PLoS Curr Outbreaks. 2014; 1–18. 10.1371/currents.outbreaks.02bc6d927ecee7bbd33532ec8ba6a25f PMC4205153 25642370

[50] Althaus CL. Ebola superspreading. Lancet Infect Dis. 2015;15: 507–508. 10.1016/S1473-3099(15)70135-0 25932579 PMC7158960

[51] Toth DJA, Gundlapalli AV, Khader K, Pettey WBP, Rubin MA, Adler FR, et al Estimates of Outbreak Risk from New Introductions of Ebola with Immediate and Delayed Transmission Control. Emerg Infect Dis J. 2015;21: 1402. 10.3201/eid2108.150170 PMC4517734 26196264

[52] Duffy S, Shackelton LA, Holmes EC. Rates of evolutionary change in viruses: patterns and determinants. Nat Rev Genet. 2008;9: 267–276. 10.1038/nrg2323 18319742

[53] Park DJ, Dudas G, Wohl S, Goba A, Whitmer SLM, Andersen KG, et al Ebola Virus Epidemiology, Transmission, and Evolution during Seven Months in Sierra Leone. Cell. 2015;161: 1516–1526. 10.1016/j.cell.2015.06.007 26091036 PMC4503805

[54] Tong Y-G, Shi W-F, Liu D, Qian J, Liang L, Bo X-C, et al Genetic diversity and evolutionary dynamics of Ebola virus in Sierra Leone. Nature. 2015;524: 93–96. 10.1038/nature14490 25970247 PMC10601608

[55] World Health Organization. WHO Ebola Situation Report (11 November 2015). [Internet]. 2015. Available: http://apps.who.int/iris/bitstream/10665/194050/1/ebolasitrep_11Nov2015_eng.pdf?ua=1&ua=1

[56] World Health Organization. WHO Ebola Situation Report (3 February 2016). [Internet]. 2016. Available: http://apps.who.int/iris/bitstream/10665/204285/1/ebolasitrep_3Feb2016_eng.pdf?ua=1&ua=1

[57] World Health Organization. Latest Ebola outbreak over in Liberia; West Africa is at zero, but new flare-ups are likely to occur. In: 14 January 2016 News Release [Internet]. 2016. Available: http://www.who.int/mediacentre/news/releases/2016/ebola-zero-liberia/en/

[58] Obilade TT. Ebola Virus Disease Stigmatization; The Role of Societal Attributes. Int Arch Med. 2015;8: 1–19.

[59] Sprecher A. Handle Survivors with Care. N Engl J Med. 2015. 10.1056/NEJMe1512928 26465064

