# Supplementary figures and images for: Potential Impact of Sexual Transmission on Ebola Virus Epidemiology: Sierra Leone as a Case Study
Source: PLoS Negl Trop Dis. 2016 May 2;10(5):e0004676. doi: 10.1371/journal.pntd.0004676 (PMC4852896; doi:10.1371/journal.pntd.0004676)

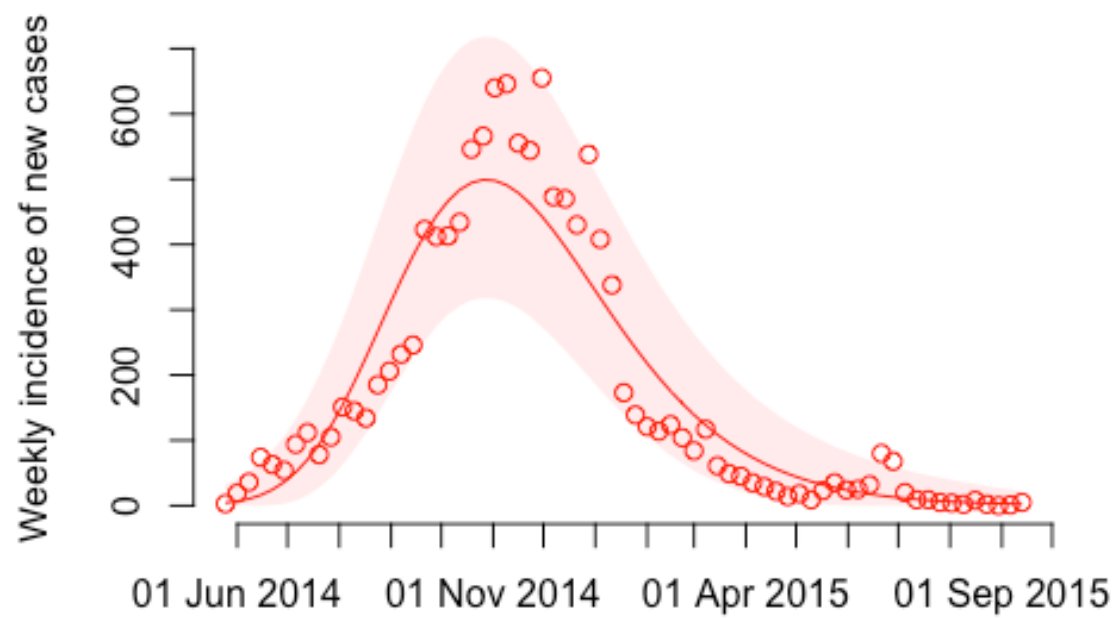

Supplement: S1 Fig — Model fit to weekly incidence of confirmed and probable cases are shown together with data from the patient database as reported by WHO (circles) [19]. The shaded area corresponds to the 95% prediction interval, assuming that the number of reported cases follows a negative binomial distribution. (PDF) [file pntd.0004676.s001.pdf]

% Contribution of  $R_0c$  to effective reproductive number

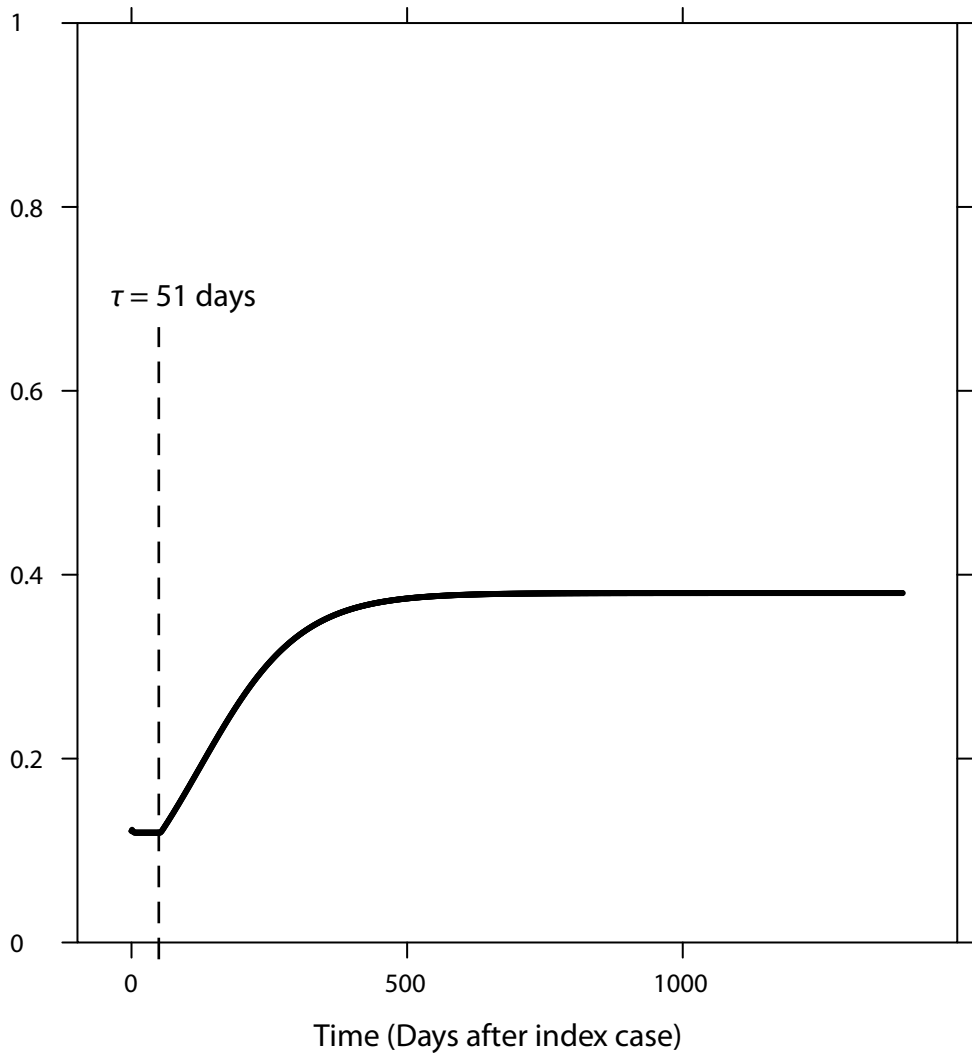

Supplement: S2 Fig — The relative contribution of R0,C to the overall reproductive number increases over the course of the epidemic, as the non-sexual transmission rate falls due to the implementation of control measures and as the number of susceptible individuals declines. The dashed line indicates the day control measures were implemented (τ = 51 days after the index case). (PDF) [file pntd.0004676.s002.pdf]

## Sensitivity Analysis

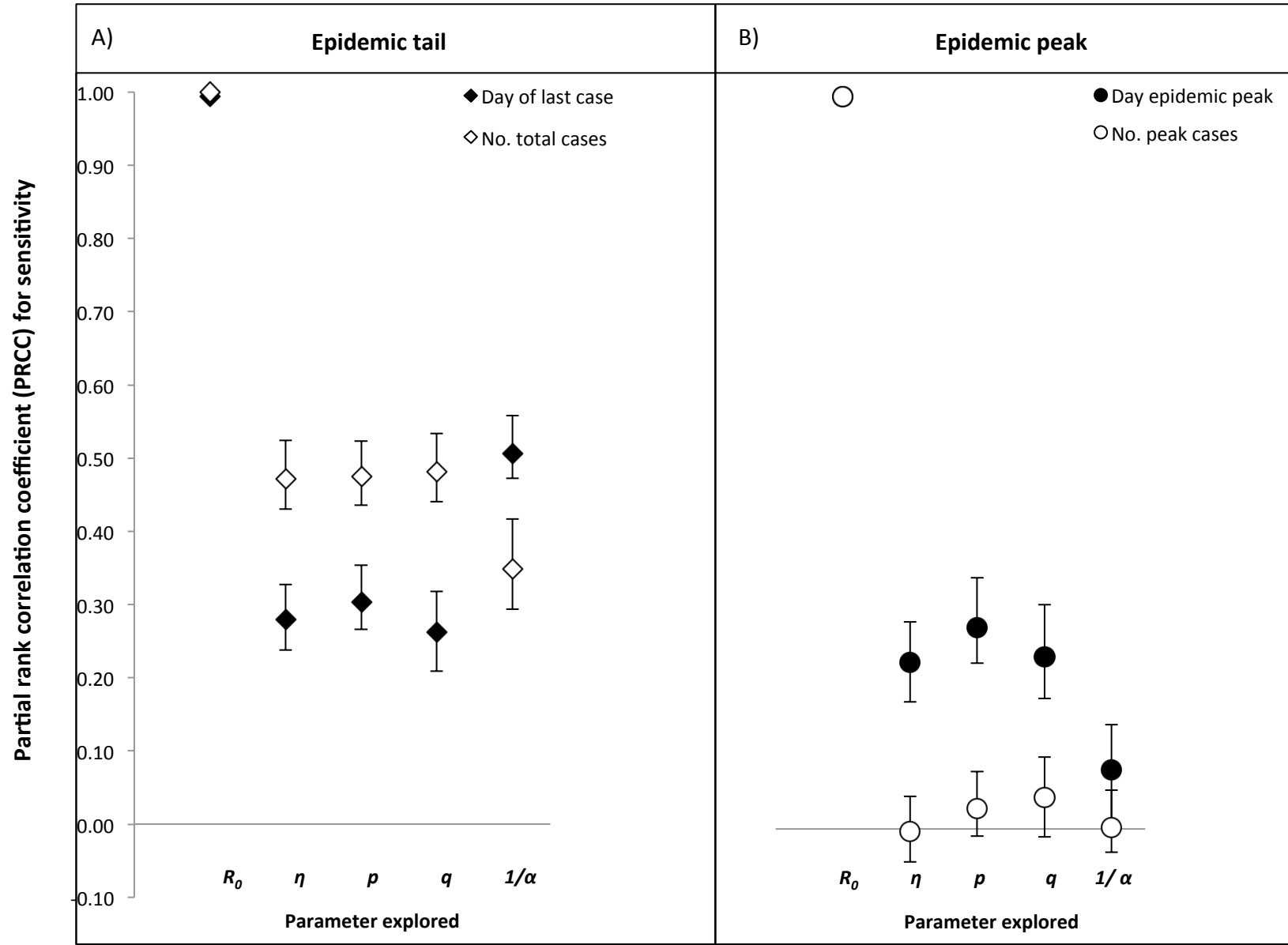

Supplement: S3 Fig — Partial rank correlation coefficients (PRCCs) and 95% confidence intervals for η (per sex act transmission probability), p (frequency of sex acts), q (proportion of the convalescent individuals who are male and sexually active), and 1/α (convalescent period), as well as the reproductive number R0. (A) Sensitivity of the duration and size of the epidemic on changes of parameters. (B) Sensitivity of the timing and daily incidence of symptomatic cases, I, at the peak of the epidemic on changes of parameters. (PDF) [file pntd.0004676.s003.pdf]
