# Supplementary material for: Potential Impact of Sexual Transmission on Ebola Virus Epidemiology: Sierra Leone as a Case Study
Source: PLoS Negl Trop Dis. 2016 May 2;10(5):e0004676. doi: 10.1371/journal.pntd.0004676 (PMC4852896; doi:10.1371/journal.pntd.0004676)
Supplement: S4 Fig — The effects of two-fold changes in convalescent period (1/ α) and in per sex act sexual transmission probability (η) on the average duration of Monte Carlo simulated Ebola virus epidemics. Box and whisker plots show the mean and variance for the length of those epidemics (number of days symptomatic cases remained in the population) which sustained 50 or more total cases (out of 1000 simulations). Statistical significance of comparisons were corrected for multiple tests following the Games-Howell method, and all are reported in Supporting Information S1 Table. Asterisks denote p-values of (*) < 0.05 and (***) <0.0001. (PDF) [file pntd.0004676.s004.pdf]

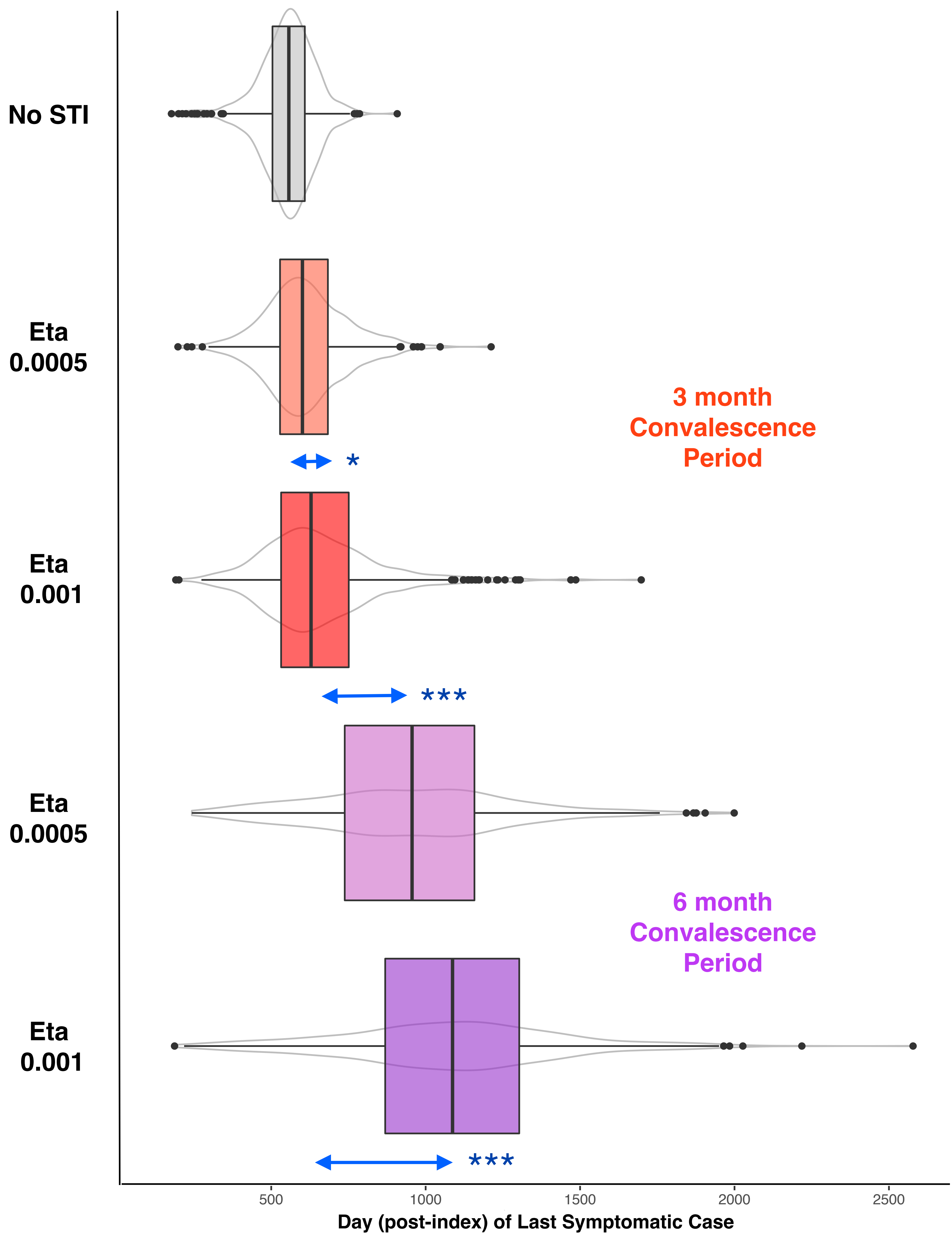
No STI
Eta 0.0005
Eta 0.001
Eta 0.0005
Eta 0.001
3 month Convalescence Period
6 month Convalescence Period
*
***
***
500
1000
1500
2000
2500
Day (post-index) of Last Symptomatic Case
