# Supplementary material for: Potential Impact of Sexual Transmission on Ebola Virus Epidemiology: Sierra Leone as a Case Study
Source: PLoS Negl Trop Dis. 2016 May 2;10(5):e0004676. doi: 10.1371/journal.pntd.0004676 (PMC4852896; doi:10.1371/journal.pntd.0004676)
Supplement: S1 Table — Pairwise effects of two-fold changes in convalescent period (1/ α) and in per sex act sexual transmission probability (η) on the average duration of Monte Carlo simulated Ebola virus epidemics. Out of 1000 simulations for each set of parameters (α and η), the mean length (number of days) of the epidemics having at least 50 total cases (n = number of simulations) is given in the diagonal. Statistical significance of each pairwise comparison is given above the diagonal (t statistic (df) and p-value) and were corrected for multiple tests using the Games-Howell method. The relative reduction in the length of the epidemic (% fewer days) is given below the diagonal. The red and blue values are those referenced in the results of the manuscript. (PDF) [file pntd.0004676.s005.pdf]

			Convalescent period (1/α)			
			3 months		6 months	
			Per sex act sexual transmission probability (η)			
1/α	η	No STI	0.0005	0.001	0.0005	0.001
	No STI	548 days<br>n=500	8.2 (895)<br>1.2e-13<br>***	10.8 (868)<br>5.5e-14<br>***	26.7 (608)<br>3.9e-10<br>***	33.7 (608)<br>3.8e-10<br>***
3 months	0.0005	-9.57%	606 days<br>n=509	2.8 (1027)<br>0.042<br>*	21.7 (701)<br>0.0e+00<br>***	29.0 (687)<br>0.0e+00<br>***
	0.001	-13.15%	**-3.96%**	631 days<br>n=529	19.7 (743)<br>0.0e+00<br>***	27.1 (723)<br>0.0e+00<br>***
6 months	0.0005	-41.39%	-35.19%	-32.51%	935 days<br>n=523	7.3 (1050)<br>6.1e-12<br>***
	0.001	-49.63%	**-44.30%**	-42.00%	**-14.06%**	1088 days<br>n=538

*p < 0.05 ** p < 0.0001
