# Supplementary material for: Potential Impact of Sexual Transmission on Ebola Virus Epidemiology: Sierra Leone as a Case Study
Source: PLoS Negl Trop Dis. 2016 May 2;10(5):e0004676. doi: 10.1371/journal.pntd.0004676 (PMC4852896; doi:10.1371/journal.pntd.0004676)
Supplement: S1 Appendix — (PDF) [file pntd.0004676.s006.pdf]

Supporting Information

Potential impact of sexual transmission on Ebola virus epidemiology: Sierra Leone as a case study

Jessica L. Abbate[1,2,3]*†, Carmen Lia Murall[4]†, Heinz Richner[1], Christian L. Althaus[5]

[1]Institute for Ecology and Evolution, University of Bern, Bern, Switzerland.

[2]UMR MIVEGEC (UMR CNRS 5290, IRD 224, UM), Montpellier, France.

[3]UMR UMMISCO (UMI 209 IRD-UPMC), Bondy, France.

[4]Max-Planck Institute for Dynamics and Self-Organization, Göttingen, Germany.

[5]Institute of Social and Preventive Medicine (ISPM), University of Bern, Bern, Switzerland.

*Direct correspondence to: jessica.abbate@ird.fr

†Authors contributed equally to this article.

December 11, 2015

S1 Appendix. Calculating R_0

At the beginning of the epidemic when the population is entirely susceptible ($S/N = 1$), the SEICR model from the main text can be written as:

$$\frac{dS}{dt} = -\beta SI - \beta_S pC,$$
$$\frac{dE}{dt} = \beta SI + \beta_S pC - \sigma E,$$
$$\frac{dI}{dt} = \sigma E - \gamma I,$$
$$\frac{dC}{dt} = (1-f)\gamma I - \alpha C,$$
$$\frac{dR}{dt} = \alpha C,$$
$$\frac{dD}{dt} = f\gamma I.$$

The model has three disease stages, exposed, E, infected, I, and convalescent, C, and thus, a vector representing these disease stages can be defined as $\mathrm{x(t)} = \begin{pmatrix} E \\ I \\ C \end{pmatrix}$. Let F be the *infection matrix* and V be the *transition matrix*. Since both I and C can transmit the virus, the matrices F and V become

$$F = \begin{pmatrix} 0 & \beta S_0 & \beta_S p \\ 0 & 0 & 0 \\ 0 & 0 & 0 \end{pmatrix} \quad \text{and} \quad V = \begin{pmatrix} \sigma & 0 & 0 \\ -\sigma & \gamma & 0 \\ 0 & -(1-f)\gamma & \alpha \end{pmatrix}.$$

The progression through disease stages can be found from $x't = Fx(t) - Vx(t)$, and thus the expected number of secondary infections is

$$\int_0^\infty Fx(t)dx = Kx(0),$$

where the *next generation matrix* is $K = FV^{-1}$ and its positive real eigenvalue is the basic reproductive number:

$$R_0 = \frac{\beta S_0}{\gamma} + \frac{(1-f)\beta_S p}{\alpha}.$$

Note that when α goes to infinity or either $\beta_s = 0$ or $p = 0$, the equation reduces to $R_0 = \frac{\beta S_0}{\gamma}$, which is the basic reproductive number in absence of sexual transmission. Therefore, the contribution of sexual transmission to the overall R_0 is simply

$$R_{0,C} = \frac{(1-f)\beta_S p}{\alpha}.$$
